# 不同病理组织学分级滤泡淋巴瘤的临床PET-CT影像特征及预后研究

**DOI:** 10.3760/cma.j.cn121090-20240208-00059

**Published:** 2024-08

**Authors:** 彤 赵, 敏 白, 蕊 王, 铭 赵, 蓉蓉 田, 军 邢, 艳梅 林, 洁 周, 凌 原

**Affiliations:** 1 山西省肿瘤医院、中国医学科学院肿瘤医院山西医院、山西医科大学附属肿瘤医院核医学PET/CT中心，太原 030013 Department of Nuclear Medicine（PET/CT）, China Shanxi Province Cancer Hospital/ Shanxi Hospital Affiliated to Cancer Hospital, Chinese Academy of Medical Sciences/Cancer Hospital Affiliated to Shanxi Medical University, Taiyuan 030013, China; 2 山西省肿瘤医院、中国医学科学院肿瘤医院山西医院、山西医科大学附属肿瘤医院血液科，太原 030013 Department of Hematology, China Shanxi Province Cancer Hospital/ Shanxi Hospital Affiliated to Cancer Hospital, Chinese Academy of Medical Sciences/Cancer Hospital Affiliated to Shanxi Medical University, Taiyuan 030013, China; 3 山西医科大学法医学院，太原 030001 School of Forensic Medicine, Shanxi Medical University, Taiyuan 030001, China

## Abstract

不同病理组织学分级滤泡淋巴瘤（FL）呈高度异质性，其生物学特性和临床管理方式存在差异。本研究回顾性分析山西省肿瘤医院161例不同病理组织学分级（1～2级、3A级、3B级）FL患者的^18^F-氟代脱氧葡萄糖（FDG）PET-CT代谢参数、临床特征及其和预后的关系。病理组织学分级1～2级93例，3A级40例，3B级28例。LDH、CD10、EZH2、c-Myc、CD37蛋白表达与病理组织学分级（1～2级、3A级、3B级）有关（*P*值均<0.05），三组间的最大标准化摄取值（SUVmax）、糖酵解总量（TLG）、肿瘤-纵隔血池标准化摄取值比（TBR）和肿瘤-肝脏标准化摄取值比（TLR）差异均有统计学意义（*P*值均<0.05）。SUVmax、肿瘤代谢体积（MTV）、TLG、TBR、TLR预测FL疾病进展最佳界值分别为8.32、201.31、2 342.55、6.56、3.52，大于界值组的患者疾病进展率增加（*P*值均<0.05）。血清β_2_微球蛋白（>2.3 µg/L）、滤泡淋巴瘤国际预后指数（FLIPI-1）评分（3～5分）、CD37蛋白表达阴性、c-Myc蛋白表达阳性、TLG（>2 342.55 g）均是影响FL患者无进展生存的独立危险因素（*HR*＝3.609、2.509、0.255、3.506、13.531，*P*值均<0.05）。^18^F-FDG PET-CT是FL病理组织学分级的有力补充，两者结合可以更好地预测FL患者的预后。

## Abstract (en)

Follicular lymphoma（FL）is highly heterogeneous with different histopathologic grades. Its biological characteristics and clinical management are different. This study retrospectively analyzed ^18^F-FDG PET-CT metabolic parameters, clinical features, and their relationship with prognosis in 161 FL patients with different histopathological grades（grade 1–2, grade 3A, grade 3B）at the Shanxi Cancer Hospital. There were 93 cases in the grade 1–2 group, 40 cases in the grade 3A group, and 28 cases in the grade 3B group. The expression of LDH, CD10, EZH2, c-Myc, and CD37 proteins was correlated with histological grade（grade 1–2, grade 3A, and grade 3B）（all *P* values<0.05）. The SUVmax, TLG, TBR, and TLR for the three groups were different（all *P* values<0.05）. The optimal thresholds of SUVmax, MTV, TLG, TBR, and TLR for predicting FL disease progression were 8.32, 201.31, 2 342.55, 6.56, and 3.52, respectively, and the rate of disease progression increased in patients with higher thresholds（all *P* value<0.05）. β_2_-MG（>2.3 µg/L）, Follicular lymphoma international prognostic index-1（FLIPI-1）score（3–5 points）, negative CD37 expression, positive c-Myc expression, and TLG（>2 342.55 g）were all independent risk factors for PFS in the FL patients（*HR*＝3.609, 2.509, 0.255, 3.506, 13.531, all *P* value<0.05）. ^18^F-FDG PET-CT is a powerful complement to FL histopathological grading and the combination of the two may better predict the prognosis of FL patients.

滤泡淋巴瘤（FL）是最常见的惰性非霍奇金淋巴瘤（NHL），呈高度异质性[1]–[2]。2022年国际临床咨询委员会公布的成熟淋巴细胞肿瘤分类[3]将FL病理组织学分级根据滤泡区中心母细胞数量分为1～2级、3A级和3B级，其中1～2级进展缓慢，3B级侵袭性较强，而3A级一直存在争议[4]。不同病理组织学分级FL的生物学特性和临床管理方式存在差异，部分患者在治疗期间会出现对免疫化疗反应不佳、早期复发进展以及发生组织学转化等情况，预后较差[5]–[6]。因此，如何早期识别这些患者、更精准地实行一线病理组织学分级分层治疗，并改善其预后是目前FL研究领域的难题和研究热点。^18^F-氟代脱氧葡萄糖（FDG）PET-CT逐渐应用于FL的诊断、临床分期、疗效评价及预后评估，但研究结果存在争议[7]。本文拟对比分析161例FL患者不同病理组织学分级的分子影像特征［最大标准化摄取值（SUVmax）、肿瘤代谢体积（MTV）、糖酵解总量（TLG）、肿瘤-纵隔血池标准化摄取值比（TBR）、肿瘤-肝脏标准化摄取值比（TLR）］和临床特征，旨在寻找影响FL患者的预后因素，探讨^18^F-FDG PET-CT及病理组织学分级预测FL预后的价值。

## 病例与方法

1. 病例：回顾性分析2016年1月至2023年3月在山西省肿瘤医院初诊并经病理证实FL患者161例，所有患者资料均包括基线（治疗前1周）^18^F-FDG PET-CT显像资料及完整的临床资料。PET-CT显像前均未接受任何治疗，且与病理组织学检查间隔时间不超过4周。排除标准为：①临床诊断不明确；②同时存在其他肿瘤、慢性疾病或传染性疾病；③淋巴瘤病灶已切除；④FL明确转化为弥漫大B细胞淋巴瘤（DLBCL）者；本研究通过山西省肿瘤医院医学伦理委员会批准（批件号：2022JC13），所有患者行^18^F-FDG PET-CT检查前均签署了知情同意书。

2. 治疗方案：161例FL患者中，一线治疗采用利妥昔单抗靶向联合化疗（R-化疗）方案79例，其中R-CHOP（R+环磷酰胺+阿霉素+长春新碱+泼尼松）方案67例，R-CVP（R+环磷酰胺＋长春新碱＋泼尼松）方案2例，BR（R+苯达莫司汀）方案10例；采用奥妥珠单抗靶向联合化疗（G-化疗）方案45例，其中G-CHOP方案31例，G-CVP方案5例，GB方案9例；单纯化学药物治疗（CHOP方案）19例，放疗4例，靶向治疗3例，免疫治疗3例，离院观察8例。

3. 显像方法：采用德国西门子Biograph mCT·s 64全身动态PET-CT扫描仪显像，^18^F由美国GE公司Minitrace医用回旋加速器生产，^18^F-FDG由化学合成模块（北京派特生物技术有限公司）自动合成，放射化学纯度>95％。患者注射^18^F-FDG前24 h内避免剧烈运动，并禁食6 h以上，检查前血糖控制在11.1 mmol/L以下。患者平静状态下按体重静脉注射^18^F-FDG 4.44～5.55 MBq/kg，1 h后排空膀胱并饮水约500 ml后行全身显像，扫描范围为颅顶至股骨上段。

4. 图像分析：由2位以上有PET-CT诊断经验的核医学医师使用syngo.via工作站处理PET和CT数据，将SUV阈值设置为患者病灶SUVmax 41％值以自动获得SUVmax、MTV、TLG，记录纵隔血池最大标准化摄取值（SUVmax-纵隔）、肝脏最大标准化摄取值（SUVmax-肝脏），并计算出TBR、TLR。

5. 随访及生存标准：采用山西省肿瘤医院病案系统及门诊电话进行随访，随访截止日期为2023年9月30日，随访时间6～92个月。研究终点为无进展生存（PFS）期，PFS期定义为从疾病确诊至疾病复发、进展、死亡或随访截止的时间。

6. 统计学处理：采用SPSS 25.0及MedCalc软件分析，符合正态分布的计量资料采用*x*±*s*表示，偏态分布的计量资料采用*M*（*Q*1，*Q*3）表示。使用受试者工作特征曲线（ROC曲线）获取SUVmax、MTV、TLG、TBR、TLR的最佳界值，组间、组内差异分析采用*χ*^2^检验、Log-rank检验、Kruskal-Wallis秩和检验，生存分析采用Kaplan-Meier法、Cox比例风险回归模型，以*P*<0.05为差异有统计学意义。

## 结果

1. 不同病理组织学分级FL与临床特征的关系：如表1所示，161例FL患者中，男87例，女74例，中位年龄53（23～85）岁。病理组织学分级1～2级93例，3A级40例，3B级28例。不同病理组织学分级在LDH、CD10、EZH2、c-Myc、CD37蛋白表达方面差异具有统计学意义（*P*值均<0.05），而在性别、年龄、Ann Arbor分期、β_2_微球蛋白（β_2_-MG）、FLIPI-1评分、FLIPI-2评分、CD5与CD8蛋白表达方面差异无统计学意义（*P*值均>0.05）。

**表1:** 161例滤泡淋巴瘤患者不同病理组织学分级的临床特征比较[例（%）]

临床特征	例数	疾病进展	病理组织学分级			*χ*^2^值	*P*值
			1～2级（93例）	3A级（40例）	3B级（28例）		
性别						1.215	0.545
男	87	26（29.9）	51（31.7）	19（11.8）	17（10.6）		
女	74	20（27.0）	42（26.1）	21（13.0）	11（6.8）		
年龄（岁）						4.740	0.094
≤60	108	28（25.9）	67（41.6）	27（16.8）	14（8.7）		
>60	53	18（34.0）	26（16.1）	13（8.1）	14（8.7）		
Ann Arbor分期						2.943	0.230
Ⅰ～Ⅱ期	41	5（12.2）	19（11.8）	13（8.1）	9（5.6）		
Ⅲ～Ⅳ期	120	41（34.2）	74（46.0）	27（16.8）	19（11.8）		
LDH（U/L）						13.466	0.001
≤248	118	26（22.0）	68（42.2）	36（22.4）	14（8.7）		
>248	43	20（46.5）	25（15.5）	4（2.5）	14（8.7）		
β_2_微球蛋白（µg/L）						0.055	0.973
≤2.3	82	13（15.9）	47（29.2）	21（13.0）	14（8.7）		
>2.3	79	33（41.8）	46（28.6）	19（11.8）	14（8.7）		
FLIPI-1评分						5.544	0.063
0～2分	106	18（17.0）	61（37.9）	31（19.3）	14（8.7）		
3～5分	55	28（17.4）	32（19.9）	9（5.6）	14（8.7）		
FLIPI-2评分						2.765	0.251
0～2分	118	27（22.9）	71（44.1）	30（18.6）	17（10.6）		
3～5分	43	19（44.2）	22（13.7）	10（6.2）	11（6.8）		
CD5蛋白表达						1.474	0.478
阴性	123	28（22.8）	72（44.7）	28（17.4）	23（14.3）		
阳性	38	18（47.4）	21（13.0）	12（7.5）	5（3.1）		
CD10蛋白表达						13.232	0.001
阴性	44	21（47.7）	16（9.9）	14（8.7）	14（8.7）		
阳性	117	25（21.4）	77（47.8）	26（16.1）	14（8.7）		
EZH2蛋白表达						6.892	0.032
阴性	37	4（10.8）	28（17.4）	4（2.5）	5（3.1）		
阳性	124	42（33.9）	65（40.4）	36（22.4）	23（14.3）		
c-Myc蛋白表达						13.152	0.001
阴性	111	23（20.7）	73（45.3）	26（16.1）	12（7.5）		
阳性	50	23（46.0）	20（12.4）	14（8.7）	16（9.9）		
CD8蛋白表达						3.220	0.200
阴性	85	29（34.1）	50（31.1）	17（10.6）	18（11.2）		
阳性	76	17（22.4）	43（26.7）	23（14.3）	10（6.2）		
CD37蛋白表达						10.324	0.006
阴性	18	12（66.7）	7（4.3）	3（1.9）	8（5.0）		
阳性	143	34（23.8）	86（53.4）	37（23.0）	20（12.4）		

**注** FLIPI：滤泡淋巴瘤国际预后指数

2. 不同病理组织学分级^18^F-FDG PET-CT代谢参数的比较：如表2所示，不同病理组织学分级的FL患者SUVmax、TLG、TBR及TLR差异均有统计学意义（*P*值均<0.05），而MTV差异无统计学意义（*H*＝0.584，*P*>0.05）。将有统计学意义的PET-CT参数进行Kruskal-Wallis秩和检验成对比较，结果显示病理组织学分级1～2级组与3A级组间，与3B级组间的PET-CT参数差异均有统计学意义（*P*值均<0.05），但3A级组与3B级组的PET-CT参数差异均无统计学意义（*P*值均>0.05）。

**表2:** 161例滤泡淋巴瘤患者不同病理组织学分级的^18^F-氟代脱氧葡萄糖（FDG）PET-CT代谢参数比较［*M*（*Q*1,*Q*3）］

^18^F FDG PET-CT 代谢参数	病理组织学分级（161例）			*H*值	*P*值
	1～2级组（93例）	3A级组（40例）	3B级组（28例）		
SUVmax	8.52（6.77，9.96）	17.40（15.46，19.98）^a^	20.32（16.24，24.07）^a^	105.430	<0.01
MTV	268.35（105.86，462.46）	202.19（79.68，429.20）	236.97（137.67，490.91）	0.584	0.747
TLG	1 539.83（459.12，2 896.70）	2 400.27（934.62，5 438.31）^a^	2 491.35（1 414.88，8 471.46）^a^	16.305	<0.01
TBR	7.09（5.71，8.79）	11.72（10.07，13.06）^a^	12.86（11.53，14.54）^a^	83.998	<0.01
TLR	3.87（3.14，4.60）	6.96（6.30，8.35）^a^	8.48（6.82，9.75）^a^	102.690	<0.01

**注** SUVmax：最大标准化摄取值；MTV：肿瘤代谢体积；TLG：病灶糖酵解总量；TBR：肿瘤-纵隔血池标准化摄取值比；TLR：肿瘤-肝脏标准化摄取值比；^a^表示3A、3B分别与1～2级组间差异有统计学意义

3. 生存分析：161例FL患者中，46例（28.57％）患者在随访期出现疾病进展，其中17例（10.56％）患者出现死亡。病理1～2级93例中22例（23.66％）进展，3A级40例中11例（27.50％）进展，3B级28例中13例（46.43％）进展。SUVmax、MTV、TLG、TBR、TLR预测FL疾病进展的最佳界值分别为8.32、201.31、2 342.55、6.56、3.52，大于界值组的患者疾病进展率增加（*χ*^2^值：10.088、32.411、35.198、11.200、10.328，*P*值均<0.05）。

单因素Cox回归分析显示，Ann Arbor分期（Ⅲ～Ⅳ期）、LDH（>248 U/L）、β_2_-MG（>2.3 µg/L）、FLIPI-1评分（3～5分）、FLIPI-2评分（3～5分）、CD5蛋白表达阳性、CD10蛋白表达阴性、c-Myc蛋白表达阳性、CD37蛋白表达阴性、SUVmax（>8.32）、MTV（>201.31 cm^3^）、TLG（>2 342.55 g）、TBR（>6.56）、TLR（>3.52）、病理组织学分级（3B级）均为FL患者预后的不良因素；因此，我们进一步将3A级的患者纳入1～2级患者中，分为1～3A级组和3B级组，Kaplan-Meier生存分析结果显示1～3A级与3B级组间PFS差异有统计学意义（*χ*^2^＝5.818，*P*＝0.016）。将单因素分析中有统计学意义的变量进行多因素Cox回归分析，结果显示β_2_-MG（>2.3 µg/L）、FLIPI-1评分（3～5分）、CD37蛋白表达阴性、c-Myc蛋白表达阳性、TLG（>2 342.55 g）均是影响FL患者PFS的独立危险因素（*P*值均<0.05）（表3）。

**表3:** 影响161例滤泡淋巴瘤患者无进展生存的多因素Cox回归分析

因素	*HR*（95%*CI*）	*P*值
β_2_微球蛋白（>2.3 µg/L）	3.609（1.698～7.669）	0.001
FLIPI-1评分（3～5分）	2.509（1.329～4.735）	0.005
CD37蛋白表达阴性	0.255（0.131～0.497）	<0.001
c-Myc蛋白表达阳性	3.506（1.890～6.503）	<0.001
TLG（>2 342.55 g）	13.531（4.796～38.174）	<0.001

**注** FLIPI：滤泡淋巴瘤国际预后指数；TLG：病灶糖酵解总量

## 讨论

2022年第5版WHO淋巴瘤分类标准对FL进行了更新[8]。但第5版WHO分类更加注重FL的发病机制，对于指导个体化治疗的意义还有待进一步观察[1]。所以本研究依旧采用第4版（修正版）FL的分类标准（1级、2级、3A级及3B级）[3]。研究发现3B级FL易转化为侵袭性淋巴瘤，与DLBCL生物学行为相似[9]–[10]。而3A级FL临床特征及临床管理存在争议[4]。因此，我们将FL（1～2级、3A级、3B级）患者均纳入研究，分析不同病理组织学分级FL的临床特征及^18^F-FDG PET-CT代谢参数特征，探讨FL患者预后的影响因素。

本研究结果显示：SUVmax、TLG、TBR、TLR在病理组织学分级各组间差异有统计学意义，说明^18^F-FDG PET-CT代谢参数SUVmax、TLG、TBR、TLR对鉴别FL不同病理组织学分级（1～2级、3A级、3B级）有一定价值，但MTV与病理组织学分级无相关性，说明不同病理组织学分级的FL的肿瘤代谢负荷无差别，分析可能原因：①FL的特征是以弥漫性淋巴结肿大为主，结外受累较少见[11]；②低级别FL患者往往也是累及全身淋巴结。研究表明^18^F-FDG PET-CT是区分3A级和低级别FL的有用辅助手段[12]。本研究发现1～2级FL患者与3A级、与3B级间的SUVmax、TLG、TBR、TLR差异均有统计学意义，但3A级与3B级的FL患者^18^F-FDG PET-CT代谢参数差异无统计学意义，提示PET-CT可以预测FL病理组织学分级，但对于3A级和3B级FL的进一步明确，仍需要根据^18^F-FDG PET-CT代谢最高部位指导临床活检。

免疫组化指标在FL患者中对于指导靶向治疗的开展和应用起到重要作用[13]，本研究结果显示CD10、EZH2、c-Myc及CD37蛋白是否表达与FL患者病理组织学分级相关，而这与以往研究结论不尽相同，因此仍需多样本、多中心研究加以证实[14]–[17]。同时有研究发现，免疫组化指标可以预测FL患者的预后：CD37蛋白表达阴性FL患者总生存较差[16]；CD10蛋白表达阴性对FL患者的长期生存无显著影响[15]；c-Myc蛋白表达阳性FL患者PFS较差[18]。本研究结果显示CD37蛋白表达阴性、c-Myc蛋白表达阳性均是影响FL预后的独立危险因素。因此，病理组织学分级与免疫组化指标相结合能够加快FL靶向、免疫治疗研究。

基线^18^F-FDG PET-CT代谢参数（SUVmax、MTV、TLG、TBR及TLR）均能很好地预测FL预后。Liang等[19]发现基线MTV、TLG均是FL患者PFS的独立预后因素，Zhou等[20]发现仅TLG与FL患者的预后显著相关，Li等[12]建立新的预后模型研究发现TLG较MTV是更稳定的预后因素。本研究同样发现TLG是FL预后的独立危险因素。这些研究之间的差异可能与MTV或TLG使用的界值方法、风险组分布以及纳入多因素统计方法有关，或是MTV与TLG具有高度相关性，同时纳入Cox多因素分析会干扰结果。Ann Arbor分期（Ⅲ～Ⅳ期）、LDH（>248 U/L）、β_2_-MG（>2.3 µg/L）、FLIPI-1/FLIPI-2评分高危组（≥3分）均能预测FL患者的预后，但仅β_2_-MG、FLIPI-1评分是FL患者疾病复发进展的独立危险因素。在病理组织学分级中，3B级FL的患者易复发进展，而3A级患者预后可能更接近于1～2级FL患者，^18^F-FDG PET-CT影像学表现与病理组织学分级出现不一致的可能原因：①病理组织学分级仅根据穿刺活检单个部位病灶而获得，不一定准确反映肿瘤异质性；②低估了部分FL患者的病理组织学分级。我国专家共识认为，应根据^18^F-FDG PET-CT提供的SUVmax最高病灶指导穿刺活检部位，以获得更为准确的病理组织学分级[1]。

综上，^18^F-FDG PET-CT是预测FL患者病理组织学分级和判断预后的有用工具。FL病理组织学分级与^18^F-FDG PET-CT代谢参数、临床病理特征指标相互补充有助于帮助临床识别FL的异质性，制定个体化治疗方案，提高患者治愈率，病理组织学分级结合^18^F-FDG PET-CT代谢参数可以更好地预测患者的预后。
